# Radiologic Findings in Cutis Laxa Syndrome and Unusual Association with Hypertrophic Pyloric Stenosis

**DOI:** 10.5812/iranjradiol.4064

**Published:** 2013-05-20

**Authors:** Mehdi Alehossein, Masoud Pourgholami, Kamyar Kamrani, Mohammad Soltani, Afshin Yazdi, Payman Salamati

**Affiliations:** 1 Advanced Diagnostic and Interventional Radiology Research Center, Tehran University of Medical Sciences, Tehran, Iran; 2 Department of Radiology, Tehran University of Medical Sciences, Tehran, Iran; 3 Department of Pediatrics, Tehran University of Medical Sciences, Tehran, Iran

**Keywords:** Cutis Laxa, Diverticulum, Pyloric Stenosis

## Abstract

Cutis laxa (CL) is a rare congenital and acquired disorder characterized by loose and redundant skin with reduced elasticity. Three types of congenital cutis laxa have been recognized. Other findings are pulmonary emphysema, bronchiectasia, hernia and diverticulosis. We describe a female neonate involved by cutis laxa syndrome and a positive family history. We focus on the radiologic findings of this case such as multiple bladder diverticulosis, GI diverticulosis and very rare accompanying hypertrophic pyloric stenosis (HPS).

## 1. Introduction

Cutis laxa (CL) is a rare congenital and acquired disorder characterized by loose and redundant skin with reduced elasticity. Three types of congenital cutis laxa has been recognized. Autosomal dominant (AD), autosomal recessive (AR) and X-linked recessive (1). The first group, autosomal dominant, is benign with a late onset skin manifestation and a subnormal life span. The second group, autosomal recessive cutis laxa (ARCL), includes type I and type II cutis laxa. Type I ARCL is characterized by early infantile pulmonary emphysema, hernia, multiple diverticula and a poor prognosis. Type II ARCL is associated with growth and developmental delay, joint laxity, peculiar face with frontal bossing, a large fontanelle and skeletal dysplasia including congenital dislocation of the hip (2-4). Finally, the third group, X-linked recessive is currently classified in the group of copper transport diseases. There are few reports of cutis laxa in the neonate with attention to radiologic findings. Cutis laxa associated with hypertrophic pyloric stenosis (HPS) is rarely reported (5, 6).

## 2. Case Presentation

A neonate girl was born at term (3300 gram) as the second child of healthy consanguine parents. The first male baby suffered from cutis laxa and died at the age of 7 months. This female neonate was referred on the second day of life from another nursery to our hospital because of respiratory distress, skin abnormality and a palpable mass in the lower abdomen. Clinical findings at admission were palpebral edema, redundant skin and reduced elasticity (Figure 1).

**Figure:**
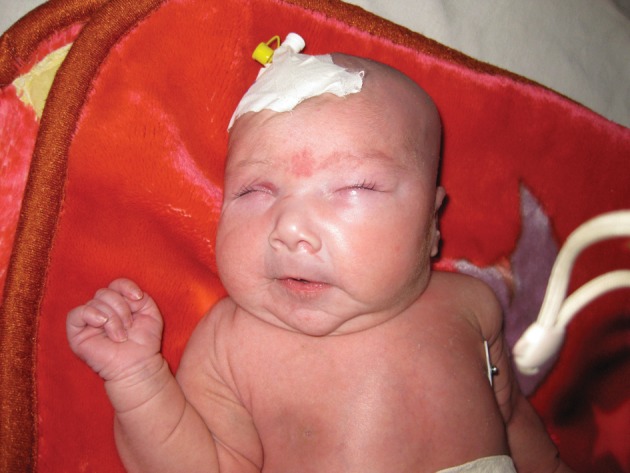
Figure 1. Photograph of the neonate demonstrates laxity of the skin and palpebral edema.

A soft mass was palpated in the left lower quadrant of the abdomen. However, thoracoabdominal plain radiography was within normal limit. Abdominal ultrasound was performed with a Sonoline G50 scanner, a 7.5-MHz sector and a 10-MHz linear-array transducer (Siemens medical solutions, Erlangen, Germany). Multiple cystic like lesions with varied sizes were noted around the bladder wall dominantly in the posterior and lateral wall. Bladder diverticulosis was suggested (Figure 2). The largest diverticulum on the left side of the bladder was responsible for the palpable soft mass in the LLQ (Figure 3).

**Figure:**
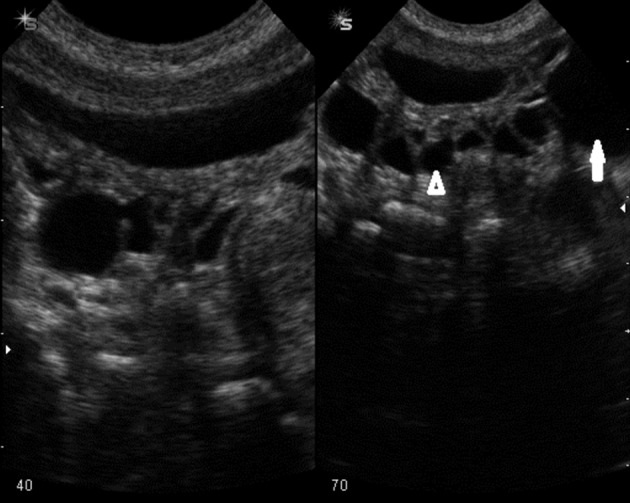
Figure 2. Bladder shows multiple muraldiverticuli with varied sizes dominantly in the posterior wall.

**Figure:**
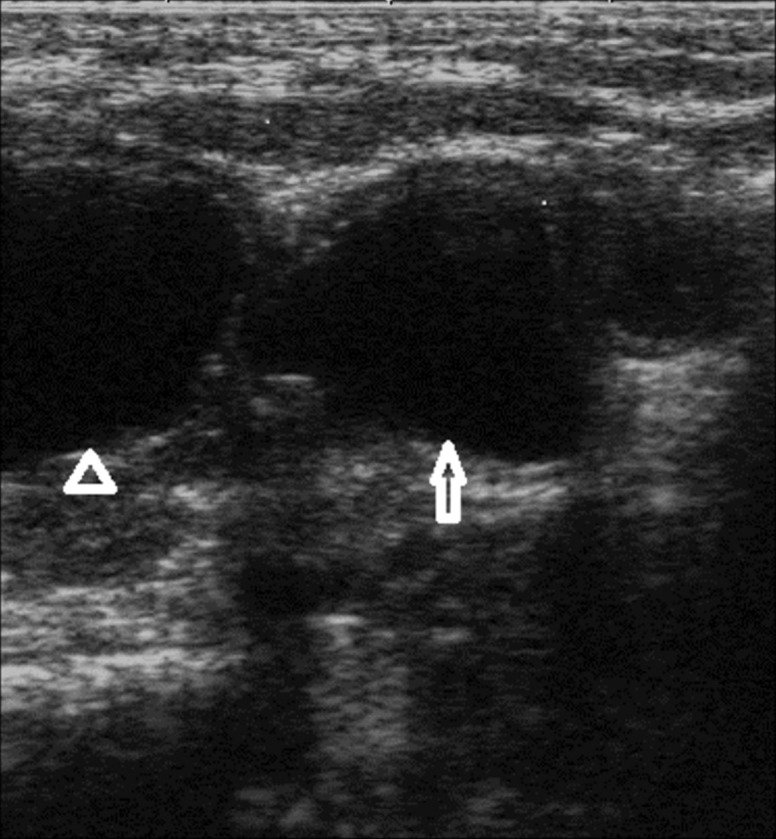
Figure 3. Close up of Figure 2. The bladder is marked with an open arrowhead. The largest left diverticulum with a left sided position is marked with an open arrow.

Abdominal US was otherwise unremarkable. Voiding cystourethrography (VCUG) confirmed bladder diverticulosis (Figure 4). Neither vesicoureteral reflux nor urethral abnormality was seen in VCUG. Echocardiography findings were atrial septal defect (ASD), ventricular septal defect (VSD), mild mitral regurgitation (MR) and mild tricuspid regurgitation (TR). Regarding the above findings, skin biopsy was performed. Microscopically, the epidermis layer was normal, but the underlying dermis revealed reduction and occasional fragment of elastic fibers. In special staining, in both papillary and reticular dermis, no inflammatory cell infiltration was seen in the dermis. Finally, marked dermal elastorrhexis and elastolysis were compatible with the clinical diagnosis of cutis laxa syndrome. Two weeks after admission, the patient was complicated with non-bilious vomiting. In the second ultrasonography, the pyloric length and width were more than 15mm and 10 mm, respectively with a 4.2 mm pyloric muscle thickening. Cervix sign was noted due to persistent hypertrophy of the pylorus. Findings were compatible with HPS (Figure 5).

**Figure:**
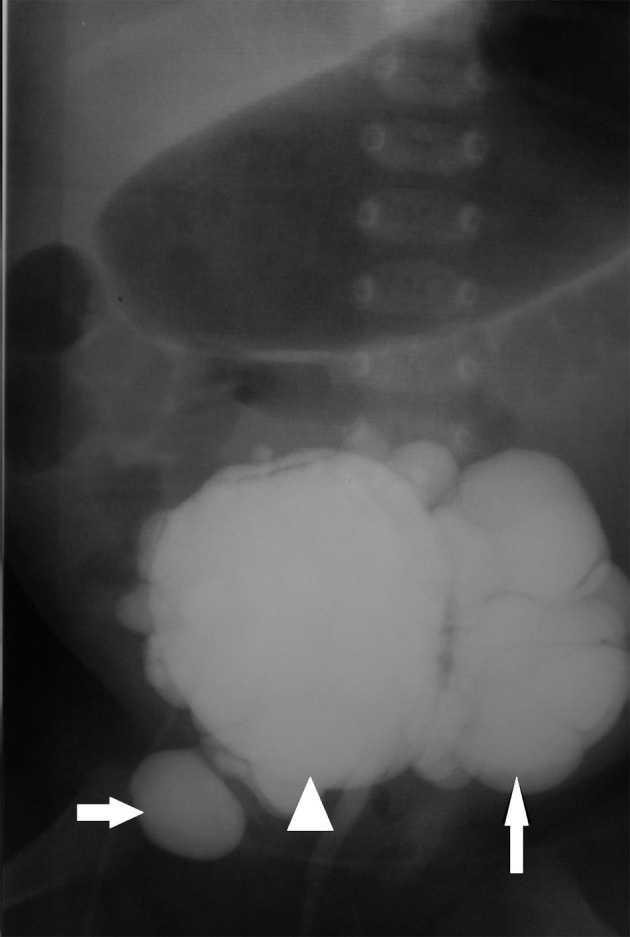
Figure 4. Voiding cystoureterogram shows multiple diverticuli with varied sizes. Arrowhead shows the bladder, the short arrow shows a diverticulum. The long arrow shows the largest diverticulum on the left side.

**Figure:**
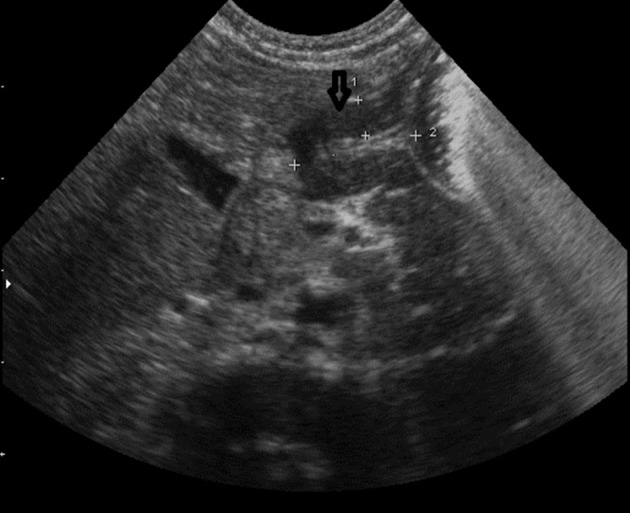
Figure 5. The classic findings of HPS demonstrated by ultrasound as abnormal muscle thickness and an elongated pyloric channel (cervix sign).

The patient had no known risk factor for this acquired disease such as erythromycin consumption. In the barium study, two diverticula were notable in the proximal and distal portions of the esophagus (Figures 6 and 7). The HPS were confirmed surgically and pyloroplastic procedure was performed. The infant was discharged from the hospital one week later in a stable condition. Outpatient follow-up of the infant showed no detectable emphysema in the conventional chest radiography until three months. However, the condition of the infant deteriorated and she died in another hospital at the age of 7 months probably from cardiopulmonary complications.

**Figure:**
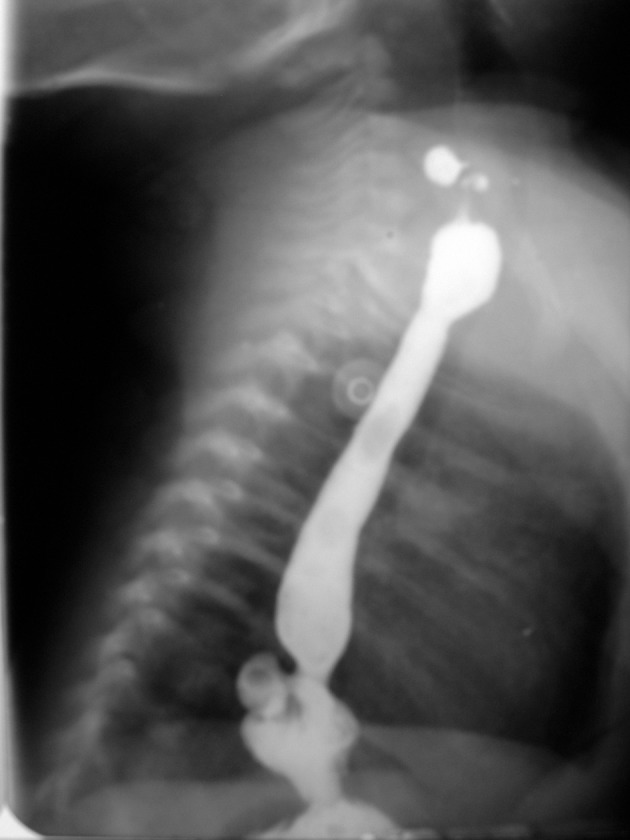
Figure 6. Esophagogram shows two dorsally-located divertcula in the cervical and lower thoracic esophagus.

**Figure:**
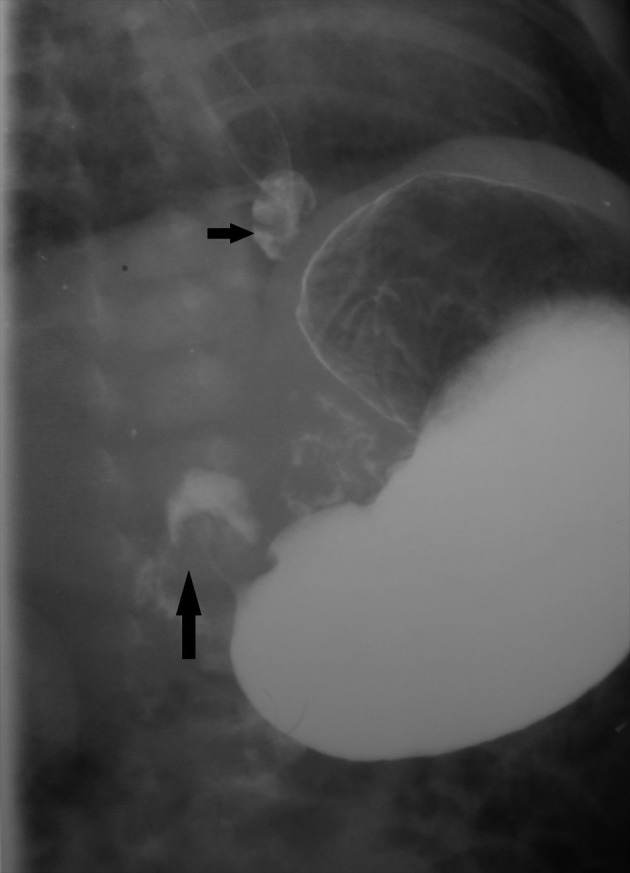
Figure 7. Upper GI series shows diverticulum in the distal third of the esophagus as the epiphrenic type (short arrow). Radiologic signs of HPS also noted as elongation of the pylorus and an umbrella sign upon the duodenal cap (long arrow).

## 3. Discussion

Cutis laxa is a heterogeneous group of congenital and rarely acquired disorders. In the congenital type, there is genetic heterogeneity with evidence of autosomal dominant, recessive or X-linked recessive inheritance. The autosomal dominant type is due to mutation in the elastic gene with a benign course and a subnormal life span. Other manifestations of this type (AD) are pulmonary artery stenosis, emphysema, bronchiectasia, hernia and genital prolapse.

The autosomal recessive cutis laxa (ARCL) includes type I (as the malignant form present at birth or shortly after birth) and type II cutis laxa (wrinkly skin syndrome and De Barsy syndrome). Type I ARCL is due to mutation of fibulin gene (5). This type is characterized by early infantile pulmonary emphysema, hernia and multiple diverticula and has poor prognosis as one of the two distinct disorders of congenital cutis laxa described first by Agha et al. (7). A deficiency in lysyl oxydase, which is closely related to wrinkly skin syndrome, has been suggested in type II ARCL. De Barsy syndrome is associated with mental retardation, short stature and corneal clouding (3-9). A “pseudoathetoid” movement disorder with onset in the second year of life appears to be a striking feature in this form of congenital cutis laxa (8).

The first metabolic disease reported in association with wrinkled, inelastic, old looking and sagging skin was Menkes disease. This inborn metabolic disease with an X-linked recessive inheritance shows systemic copper deficiency dominantly in the brain (10). The X-linked recessive (also called Ehlers-Danlos type IX syndrome or mild Menkes syndrome) has been described due to ATP7A deficiency. Unlike Ehlers-Danlos syndrome, the skin in cutis laxa, although loose, is not hyperextensible (8).

Acquired cutis laxa develops spontaneously or after a febrile illness, inflammatory skin diseases such as lupus erythematosus or erythema multiform, amyloidosis, urticaria, angioedema and hypersensitivity reactions to penicillin (11). A case of a 27-year-old female patient who developed severe pulmonary and cardiac involvement in adulthood probably suffering from the acquired type of cutis laxa is reported (12). In another study, cutis laxa syndrome was responsible for Zenker and epiphrenic diverticula (5). Gastrointestinal diverticulosis is a frequent finding in ARCL type I, but it is also seen in Ehlers-Danlos syndrome and incidentally in Noonan syndrome and multiple endocrine neoplasia type IIa and IIb.

Our presented case was a neonate with skin laxity, respiratory distress, cardiac anomalies (ASD, VSD, MR, TR), bladder diverticulosis, GI diverticulosis and hypertrophic pyloric stenosis. Bladder diverticula could be primary (Hutch, Urachal or other locations), secondary to obstruction (posterior urethral valve), neurogenic dysfunction, iatrogenic or associated with other conditions (Prune belly disease, Williams syndrome, Ehlers-Danlos, Menkes syndrome and cutis laxa) (13).

Concomitant existence of parent consanguinity, early death in affected siblings and diverticulosis implies that this case maybe an ARCL type I.

Hypertrophic pyloric stenosis is an acquired disorder more common in boys and probably first -born babies. There are some predisposing factors such as erythromycin consumption or prolonged pylorospasm. Genetic factors may also be important since it is more common in babies with an affected parent or sibling. There are some reports of CL complicated with HPS similar to our case (5, 6). To look for any association between CL and HPS, it is highly suggested to focus on further CL cases complicated with HPS.

## References

[1] Genevieve D, Baumann C, Huber C, Faivre L, Sanlaville D, Bodemer C (2004). A novel form of syndromic cutis laxa with facial dysmorphism, cleft palate, and mental retardation.. J Med Genet..

[2] Nesibe A, Fikriye S, Muhsin S, Melda Ç (2002). Autosomal Recessive Form of Congenital Cutis Laxa: More Than the Clinical Appearance.. Pediatr Dermatol..

[3] Mauskar A, Shanbag P, Ahirrao V, Nagotkar L (2010). Congenital cutis laxa.. Ann Saudi Med..

[4] Van Maldergem L, Vamos E, Liebaers I, Petit P, Vandevelde G, Simonis-Blumenfrucht A (1988). Severe congenital cutis laxa with pulmonary emphysema: a family with three affected sibs.. Am J Med Genet..

[5] Lin DS, Chang JH, Liu HL, Wei CH, Yeung CY, Ho CS (2011). Compound heterozygous mutations in PYCR1 further expand the phenotypic spectrum of De Barsy syndrome.. Am J Med Genet A..

[6] Beighton P (1972). The dominant and recessive forms of cutis laxa.. J Med Genet..

[7] Agha A, Sakati NO, Higginbottom MC, Jones KL, Bay C, Nyhan WL (1978). Two forms of cutis laxa presenting in the newborn period.. Acta Pædiatrica..

[8] Patton MA, Tolmie J, Ruthnum P, Bamforth S, Baraitser M, Pembrey M (1987). Congenital cutis laxa with retardation of growth and development.. J Med Genet..

[9] Kivuva Emma C, Parker Michael J, Cohen Marta C, Wagner Bart E, Sobey Glenda (2008). De Barsy syndrome: a review of the phenotype.. Clin Dysmorphol..

[10] Miski M, Dorus K, Thatjana G, Uwe K, Rona W, Eva M (2011). Metabolic cutis laxa syndromes.. J. Inherit. Metab. Dis..

[11] Dhale SN, Rathod AD, Sonawane S (2012). A case report of cutis laxa.. Bombay Hospital J..

[12] Shehzad A, Fareeha A, Qaseem MK (2010). Cutis laxa with systemic involvement.. J Pakistan Ass Derma..

[13] Boechat MI, Lebowitz RL (1978). Diverticula of the bladder in children.. Pediatr Radiol..

